# Further analysis of p300 in mediating effects of Butyrate in Colorectal Cancer Cells

**DOI:** 10.7150/jca.47160

**Published:** 2020-08-08

**Authors:** Michael Bordonaro

**Affiliations:** Department of Medical Education, Geisinger Commonwealth School of Medicine, 525 Pine Street, Scranton, PA 18509, USA.

**Keywords:** CBP, p300, Wnt, butyrate, colorectal cancer

## Abstract

Butyrate, a product of dietary fiber, hyperactivates Wnt signaling in colorectal cancer (CRC) cells; this activity of butyrate is causally associated with the induction of apoptosis, and the repression of proliferation, in these cells. However, CRC can develop despite a high fiber diet; hence, butyrate resistance likely occurs during colonic neoplasia. To evaluate the mechanisms of butyrate resistance, HCT-116 CRC cells were previously made resistant to butyrate (HCT-R cell line); I observed that butyrate resistance in HCT-R cells is accompanied by repressed Wnt hyperactivation. CBP and p300 competitively bind to the Wnt signaling factor beta-catenin; CBP-Wnt activity is associated with proliferation, while p300-Wnt activity is associated with differentiation and apoptosis. While butyrate sensitive HCT-116 cells express p300, butyrate resistant HCT-R cells do not. Further, HCT-116 p300 knockout cells exhibit butyrate resistance, and restoration of p300 expression in these cells enhances butyrate sensitivity. Thus, p300 activity is a mediator of butyrate sensitivity in HCT-116-derived cell lines. In the present study, YH249, a pharmacological inhibitor of the p300-beta-catenin association, was utilized to more specifically evaluate the role of p300-Wnt signaling in butyrate responsiveness. Unexpectedly, YH249 potentiates butyrate-induced effects on apoptosis and cell proliferation in HCT-116 cells; in addition, potential off-target, p300-independent, effects of YH249 on butyrate-induced Wnt hyperactivation were observed. SW620 metastatic CRC cells express p300, but do not exhibit association of p300 with beta-catenin. Thus, I hypothesized that SW620 cells can be made butyrate resistant without loss of p300 expression, while butyrate resistance would still be associated with repressed Wnt hyperactivation; this hypothesis was confirmed. Thus, the data *in toto* suggest that while p300-Wnt activity is an important effector of butyrate sensitivity in some CRC cell lines, other, p300-independent pathways influencing butyrate sensitivity must also exist.

## Introduction

The dietary fiber product butyrate, a histone deacetylase inhibitor (HDACi) hyperactivates Wnt signaling and this is causally associated with the induction of apoptosis and the repression of cell proliferation in colorectal cancer (CRC) cells 1,2. The histone acetylases CBP and p300 bind to beta-catenin in a competitive manner, triggering two different branches of Wnt activity 3. CBP-Wnt activity is associated with proliferation and p300-Wnt activity is associated with differentiation and apoptosis 3-6. A pharmacological agent, ICG-001, inhibits CBP-Wnt activity 3 and I have shown cooperative and antagonistic effects of this agent with butyrate upon cotreatment of CRC cell lines 4. CRC can develop despite a high fiber diet; hence, butyrate resistance may occur during colonic neoplasia. Since it is likely this butyrate resistance contributes to CRC that occurs within the context of a high fiber diet, understanding the mechanisms underlying resistance is important for enhancing the efficacy of fiber/butyrate as a chemopreventive agent against CRC.

I previously demonstrated that HCT-116 CRC cells can be made resistant (i.e., suppressed apoptosis and maintained cell growth) to a physiologically relevant concentration (5 mM) of butyrate through exposure to this agent (butyrate resistant HCT-R cell line); in these cells, resistance is accompanied by repressed Wnt hyperactivation 2. HCT-R cells are also cross-resistant to clinically relevant HDACis that are structurally unrelated to butyrate 2; hence, the phenomenon of butyrate resistance has implications not only for chemoprevention via butyrate but also for chemotherapeutic applications utilizing other HDACi agents.

While butyrate sensitive HCT-116 cells express p300, butyrate resistant HCT-R cells do not 5, and I have shown that HCT-116 cells in which p300 expression has been knocked out exhibit butyrate resistance 5,6. Further, restoration of p300 expression in these HCT-116 p300 knockout cells enhances butyrate sensitivity 6. These findings strongly suggest that p300 is a mediator of butyrate sensitivity in HCT-116 cells and cell lines derived from the HCT-116 line (e.g., HCT-R, p300 knockout). However, it is possible that not all CRC cell lines would exhibit the same association between p300 expression and butyrate sensitivity. For example, SW620 cells are a metastatic CRC line and although these cells express p300, they do not exhibit association of p300 with beta-catenin 4. Thus, it is reasonable to hypothesize that SW620 cells can be made butyrate resistant without loss of p300 expression. However, I would still expect that butyrate resistance in SW620 cells would be associated with inhibition of butyrate-induced Wnt hyperactivation, given the close association of Wnt hyperactivation and effects of butyrate in CRC cells 1,2.

While the findings with HCT-116 cells demonstrated the importance of p300 in that line, they did not definitively demonstrate that it is the association of p300 with beta-catenin, and hence, p300-Wnt signaling, that is specifically mediating butyrate resistance. To evaluate this in the present study, I utilized YH249, a pharmacological inhibitor targeting the p300-Wnt signaling pathway through inhibition of the p300-beta-catenin association 7. Unexpectedly, YH249 potentiates butyrate-induced effects on apoptosis and cell proliferation. This finding may be due to off-target, p300-independent effects of YH249 on butyrate-induced Wnt hyperactivation observed in HCT-116 cells. The possibility that p300-independent pathways to butyrate resistance exist was then confirmed by the creation of SW620 cells resistant to 5 mM butyrate; these cells do not downregulate p300 expression. Thus, these data suggest that p300-independent pathways influencing butyrate sensitivity exist in addition to the previously demonstrated p300-dependent modulation of butyrate sensitivity. Further, although in my hands YH249 demonstrated unexpected, possibly off-target, effects, it potentiated the ability of butyrate to induce apoptosis and repress cell proliferation and therefore may have potential clinical applications.

## Materials and Methods

### Cells, plasmids, transfection, luciferase assay

HCT-116 and F5 p300 knockout cells were kindly provided by Dr. C. Caldas via Cancer Research UK; SW620 cells were from ATCC. Wnt reporters pTOPFLASH and pFOPFLASH were obtained from Dr. H. Clevers; pRLTK from Promega. Transfections and luciferase assays were performed as previously described 1,2,4-6.

### Reagents

Butyrate was obtained from Sigma, dissolved in purified water, filter sterilized, and used at 5 mM concentration. YH249 was synthesized by Reagency (South Yarra, Australia), with DMSO (Sigma) as the vehicle.

### Apoptosis and proliferation

Apoptosis was measured using the caspase 3/7 GLO kit (Promega), as previously described 4-6. Cell proliferation was measured with the QuickCell Proliferation Kit (Biovision) as previously described 4-6.

### Western blotting

Western blotting was performed as previously described 1,2,4-6; band images were adjusted for brightness and contrast to enhance visualization.

### Statistics

Student's T-test was utilized, with statistical significance set at *P <* 0.05.

## Results

I first ascertained if pharmacological inhibition of the p300-beta-catenin association, and, hence, repression of p300-Wnt activity, induces butyrate resistance in HCT-116 cells similar to that observed with knockout of p300 6. Thus, YH249, reported to specifically interfere with the association of p300 with beta-catenin 7 was utilized to repress p300-Wnt signaling activity.

**Figure 1:**
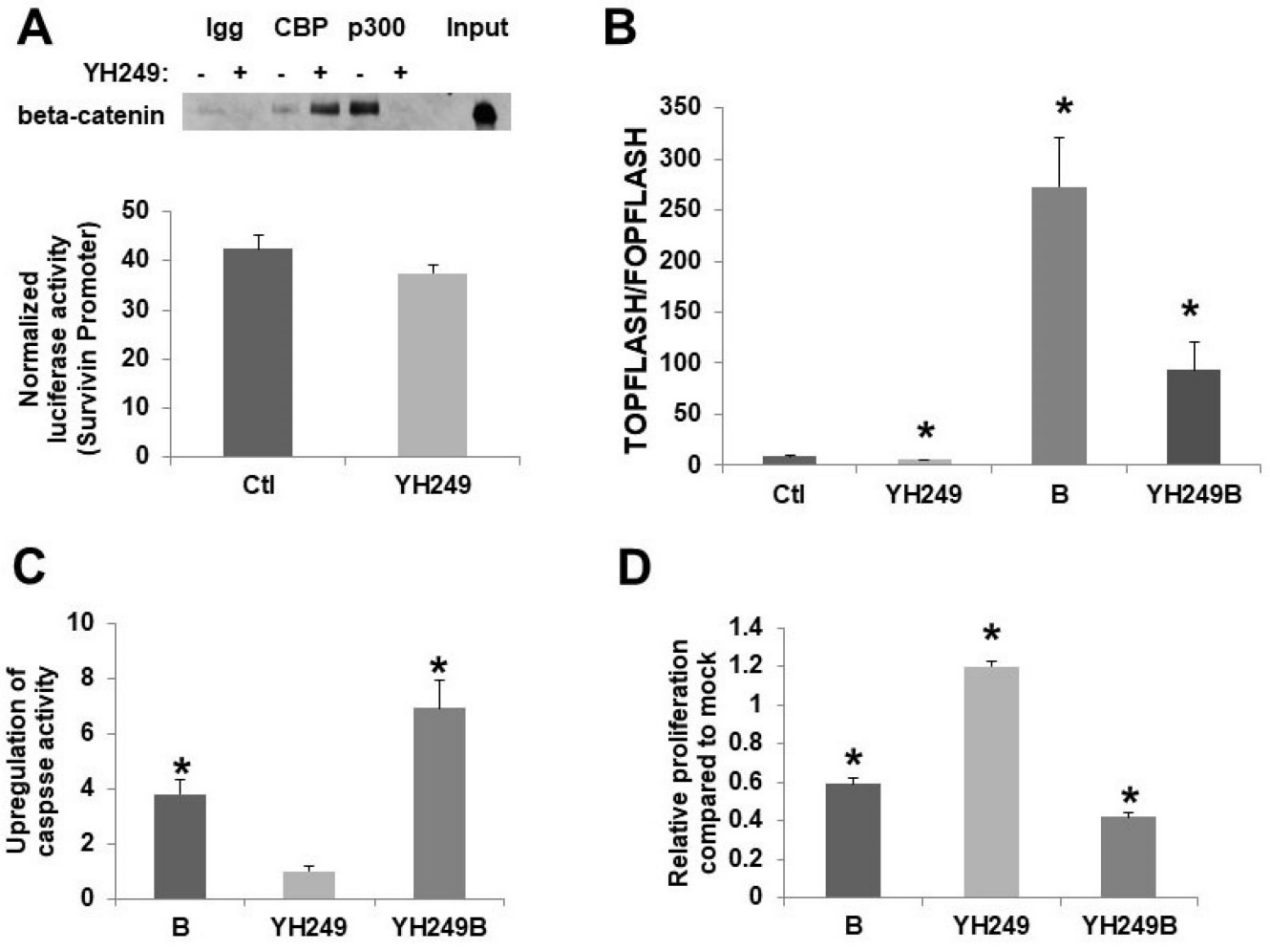
** Effects of YH249 on HCT-116 CRC cells.** (A, top) Coimmunoprecipitation of beta-catenin using CBP or p300 antibodies from nuclear lysates of HCT-116 CRC cells treated with 30 μM YH249, performed as previous described for ICG-001 treatment 4, but using with 200 μg nuclear lysate per sample as recommended for YH249 experiments 7. (A, bottom) Lipofectamine was used to transfect survivin expression vector 7 or pGL3-Control, with pRLTK, in HCT-116 cells. Cells were mock treated or treated with 30 uM YH249 for 17.5 hr. (B) TOP/FOPFlash transfections were performed as described in 1,2,4. Cells were mock treated or treated with 30 μM YH249 and/or 5 mM butyrate for 17.5 hr. Data are from three separate experiments. Bars, SDs. * = statistical significance. (C,D) Cells were mock treated or treated with 30 μM YH249 and/or 5 mM butyrate for 24 hr and caspase 3/7 activity assays (C) or cell proliferation assays (D) were performed as described in 4-6. For both the caspase and proliferation assays, data are from three separate experiments. Bars, SDs. * = statistical significance.

To confirm the reported effects of YH249 on CBP vs.p300 association with beta-catenin 7, I performed coimmunoprecipitation. I utilized 30 µM YH249; this concentration was chosen because preliminary experiments (data not shown) demonstrated that this was the lowest concentration to efficiently repress butyrate-induced Wnt hyperactivation. My findings are consistent with that previously observed 7; p300-beta-catenin binding was suppressed while CBP-beta-catenin binding was increased (Fig. 1A top). This increase is consistent with findings 3,7 that show that when the binding of CBP or p300 to beta-catenin is inhibited, the binding of the other is increased since they compete for the same binding site on beta-catenin. I also utilized a survivin promoter luciferase reporter to assess the specificity of YH249; survivin is a CBP-Wnt target gene not upregulated by p300 3,7. As expected, YH249 does not repress survivin promoter activity (Fig. 1A bottom), consistent with a lack of effect of YH249 on CBP-Wnt activity.

Basal and butyrate-induced Wnt activity in HCT-116 cells are repressed by YH249 (Fig. 1B). There is a one-third decrease in basal Wnt activity (*P <* 0.03), consistent with findings suggesting that p300-Wnt activity is a minor fraction of the total Wnt activity in these cells 4,5; further, YH249 decreased the fold-upregulation of Wnt activity by butyrate from 32.8-fold to 16.3-fold (*P <* 0.05). Since downregulation of p300 promotes butyrate resistance 4-6, I hypothesized that YH249 treatment would repress effects of butyrate on apoptosis and cell proliferation by inhibiting p300-mediated Wnt signaling. However, YH249 potentiated the effects of butyrate on both the induction of apoptosis (*P <* 0.001) (Fig. 1C) and repression of cell proliferation (*P <* 0.001) (Fig. 1D).

**Figure 2:**
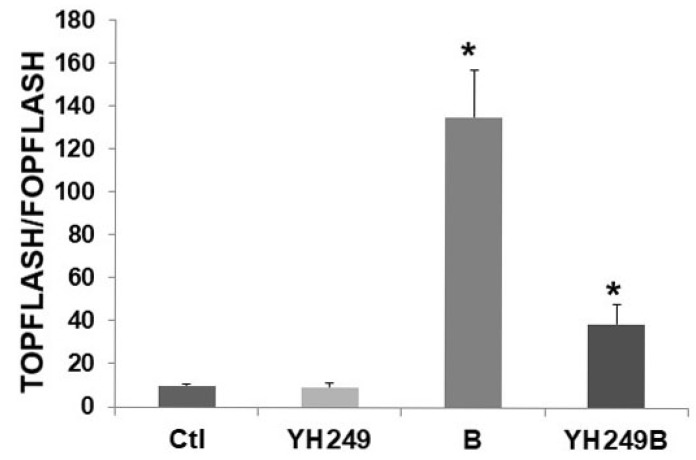
** Possible off-target effects of YH249 on HCT-116 p300 knockout cells.** Transfection, cell treatments, and luciferase assay were performed as described for Fig. 1B. Data are from three separate experiments. Bars, SDs; *statistical significance.

Since these latter findings were unexpected, I evaluated possible off-target effects of YH249 7 in F5 p300 knockout HCT-116 cells [6 and refs. therein]. Since these cells do not express p300, one would not expect effects of YH249 in these cells if the agent was acting solely through the p300-Wnt pathway. As expected, there was no change in basal Wnt activity (Fig. 2), unlike what was observed for HCT-116 cells (Fig. 1B). This suggests that while YH249 targets p300-Wnt activity in HCT-116 cells that express p300, it does not affect CBP-Wnt activity in F5 cells that lack p300. However, Wnt hyperactivation in YH249-treated F5 cells is inhibited (13.3-fold to 4.1-fold, *P <* 0.001), demonstrating that YH249 affects butyrate-induced Wnt hyperactivation independent of p300.

**Figure 3:**
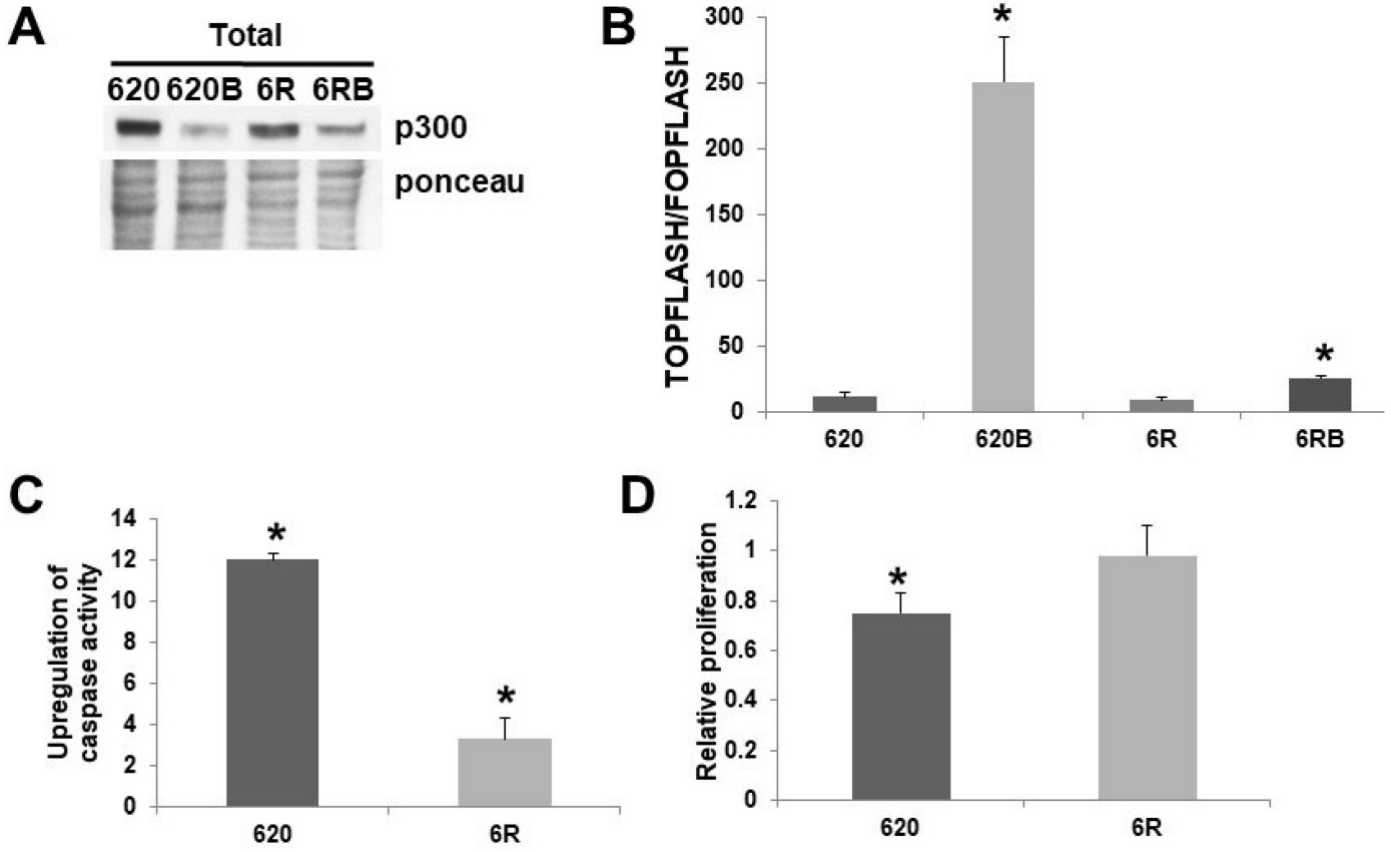
** Role of p300 in butyrate resistance in SW620 cells.** (A) Western blot data of total protein (100 μg) from untreated and butyrate (B) treated wild-type SW620 (620) and 620R (6R) CRC cells probed with an anti-p300 polyclonal antibody (Santa Cruz). To accurately identify the high molecular weight p300 band, a low percentage (6%) gel is used with long run times; therefore, ponceau staining was utilized as the loading control. (B) Butyrate resistance inhibits Wnt hyperactivation by butyrate. Wild-type SW620 (620) and butyrate-resistant 620R (6R) were transfected with the TOPFlash/FOPFlash reporter vectors and with the pRLTK normalization control. Cells were mock treated or treated with 5 mM butyrate (B) for 17.5 hr and Wnt activity (T/F) was measured as previously described 1,2,4-6. Bars, SDs; *statistical significance. C) Apoptosis measured in SW62020 and 620R cells, as described in 4-6. Data are from three separate experiments. Bars, SDs. * = statistical significance. (D) Cell proliferation was measured in SW620 (620) and 620% (6R) cells, treated or treated with 5 mM butyrate (B) for 24 hr; cell proliferation (5,000 cells/well) was measured as previously described 4-6. Data are from three separate experiments. Bars, SDs. * = statistical significance.

While I had previously generated SW620 cells resistant to 3 mM butyrate 8, for the first time I present SW620 cells resistant to 5 mM butyrate, a level equivalent to the previously reported HCT-R line 2, which I have shown is deficient in p300 expression 5. Thus, SW620 cells were made resistant to exposure to a physiologically relevant concentration of butyrate (5 mM) by constant exposure to increasing concentrations of this agent. Butyrate resistant SW620 cells (620R) cells express p300 (Fig. 3A), unlike HCT-R cells 5. I compared SW620 and 620R cells with respect to Wnt activity, apoptosis, and proliferation. Thus, I measured Wnt activity, apoptosis, and proliferation, in the presence or absence of 5 mM butyrate. 620R cells exhibit repressed Wnt hyperactivation (Fig. 3B) when exposed to 5 mM butyrate. This difference in fold-induction between the cell lines was statistically significant (*P <* 0.03), as was the difference between the overall levels of Wnt activity measured in the presence of butyrate (*P <* 0.001). These findings indicate that the hyperactivation of Wnt signaling by butyrate is sharply repressed in the butyrate-resistant 620R cell line. SW620 cells were very sensitive to the effects of butyrate on upregulating apoptosis (Fig. 3C); thus a 12-fold induction was observed (*P <* 0.001). In contrast, for 620R cells, only a 3.3-fold induction was observed (*P <* 0.01); this difference in fold-induction between the two cell lines was highly significant (*P <* 0.001). Treatment with butyrate reduced SW620 cell proliferation by 25% after 24 hr (*P <* 0.005), while for 620R cells, proliferation was unchanged (Fig. 3D); this difference was statistically significant (*P <* 0.05).

## Discussion

The data presented here (Figs. 1 and 2) suggest that YH249 does not have off-target effects on basal Wnt activity, but does have p300-independent, possibly off-target, effects on butyrate-induced Wnt activity. Regardless, YH249 cooperates with butyrate to induce CRC cell apoptosis and repress proliferation; thus, derivatives of this agent may have future clinical utility, similar to the CBP-Wnt inhibitor ICG-001 3. Given that YH249 alone resulted in a small increase in CRC cell proliferation (Fig. 1D), possibly due to as of yet unknown off-target effects, possible use of YH249 as a therapeutic agent should be restricted to the context of a high fiber diet (i.e., butyrate) and/or cotreatment with clinically relevant HDACis.

The mechanisms whereby YH249 exerts these p300-independent effects remain to be determined. The induction of butyrate resistance observed with abrogation of p300 expression and reversed by p300 re-expression 6 is not observed with YH249 (which in fact potentiates the effects of butyrate). This suggests that either downregulation of p300 expression has effects on CRC cell physiology independent of p300-Wnt signaling or that, in my hands, the off-target, p300-independent effects of YH249 influence CRC cell physiology to a greater extent than the p300-Wnt-targeted effects of this agent.

Metastatic SW620 cells selected to be resistant to 5 mM butyrate exhibit properties similar to that of butyrate resistant HCT-116 primary CRC cells. Thus, Wnt hyperactivation is inhibited (Fig. 3B), and this is associated with a suppression of butyrate-induced apoptosis (Fig. 3C), and greater proliferation in the presence of butyrate (Fig. 3D). Unlike butyrate resistant HCT-116 cells (HCT-R) 5, butyrate resistant SW620 cells (620R) express p300 (Fig. 3A). This is likely due to the fact that SW620 cells normally exhibit minimal to no association between p300 and beta-catenin 4, so there is no selective pressure to abrogate p300 expression in order for the cells to be butyrate resistant. The expression of p300 is decreased in both wild-type SW620 cells that are sensitive to the effects of butyrate and in butyrate resistant 620R cells; thus, it is unlikely that downregulation of p300 is strongly associated with butyrate resistance in the 620R line.

**Figure 4:**
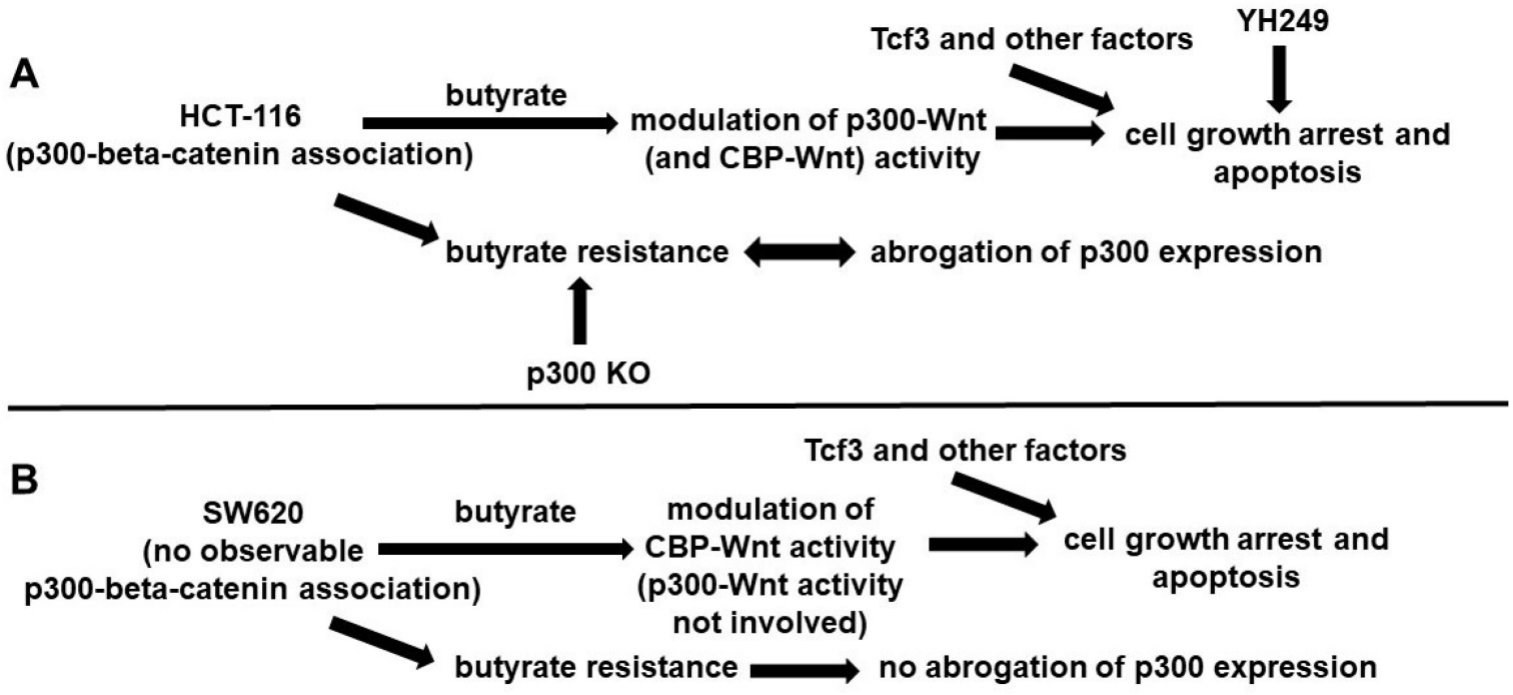
** Possible roles of p300 in CRC cell lines.** (A) In HCT-116 cells that exhibit p300-beta-catenin association, we posit that butyrate affects both p300- and CBP-Wnt activities, influencing cell growth arrest and apoptosis (in conjunction with Tcf3 and other factors). YH249 also affects these outcomes, possibly through off-target effects as well as through inhibition of p30-Wnt signaling. Butyrate resistant HCT-116 cells exhibit abrogation of p300 expression that likely influences butyrate resistance, since p300 knockout (KO) cells exhibit butyrate resistance. (B) SW620 cells differ from HCT-116 cells in that the SW620 line exhibits no observable p300-beta-catenin association. Thus, we posit that in these cells, p300-activity is not involved in the effects of butyrate, reflected in that p300 expression is still observed in butyrate resistant SW620 cells.

These findings suggest that while p300 activity may be important for the development of butyrate resistance in (at least some) CRC cell lines, it is not absolutely required, and that p300-independent mechanisms also play a role (Fig. 4). Included in these p300-dependent mechanisms maybe over-expression of Tcf3 and various cell cycle factors 9. Given that overexpression of p300 has been associated with improved prognosis for disease-free survival of CRC patients 10, understanding the role of p300 in CRC development, prevention, and treatment will be clinically important.

## Limitations and Future Studies

This short research communication is a preliminary study and thus has several limitations. Future work should evaluate how YH249 affects CBP and p300 binding to beta-catenin, as well as histone acetylation, in CRC cells exposed to butyrate. It will also be necessary to utilize ICG-001 and/or CBP knockdown to further ascertain the possible role of CBP-Wnt activity in the off-target effects of YH249, although the survivin reporter data (Fig. 1A) makes CBP involvement unlikely. Further, only two cell lines were utilized in the present study. A wider range of CRC lines, and their butyrate resistant counterparts (that need to be generated), should be evaluated before *in vivo* murine studies, which are necessary before clinical implications can be considered, can be conducted.

## Abbreviations

CRC: colorectal cancer
HDACi: histone deacetylase inhibitor
CBP: CREB binding protein
KO: knockout
